# Supplementary material for: Sorting at embryonic boundaries requires high heterotypic interfacial tension
Source: Nat Commun. 2017 Jul 31;8:157. doi: 10.1038/s41467-017-00146-x (PMC5537356; doi:10.1038/s41467-017-00146-x)
Supplement: Supplementary file 2 — Supplementary Software 1 [file 41467_2017_146_MOESM2_ESM.zip › PottsModel/SrcPottsModel/doc/model/package-tree.html]

model Class Hierarchy


JavaScript is disabled on your browser.


Skip navigation links


- Overview
- Package
- Class
- Use
- Tree
- Deprecated
- Index
- Help

- Prev
- Next

- Frames
- No Frames

- All Classes

# Hierarchy For Package model

Package Hierarchies:

- All Packages

## Class Hierarchy

- java.lang.Object
  - mvc.AObservable (implements mvc.IObservable)
    - model.Cell (implements model.KnowsConstants)
      - model.SquareCell
    - model.Lattice (implements java.lang.Iterable<T>, model.KnowsConstants, mvc.Observer)
  - model.AreaEnergyFunction (implements model.EnergyFunction)
  - model.CartesianCoordinates (implements model.Coordinates)
  - model.CellSet (implements java.lang.Iterable<T>)
  - model.Constants
  - model.Constants.ParameterManager
  - model.EnergyManager
  - model.FiniteVector (implements model.Vector)
  - model.HalfLine (implements model.Vector)
  - model.HexagonPositionManager (implements model.IPositionManager)
  - model.InterfacialTensionEnergyFunction (implements model.EnergyFunction)
  - model.Lattice.PixelIterator (implements java.util.Iterator<E>)
  - model.LatticeUtilities
  - model.Pair<A,B>
  - model.PerimeterEnergyFunction (implements model.EnergyFunction)
  - model.Pixel (implements model.KnowsConstants)
  - model.PixelInteractionEnergyFunction (implements model.EnergyFunction)
  - model.SquarePositionManager (implements model.IPositionManager)
  - model.VectorTests
  - model.VectorUtils

## Interface Hierarchy

- model.Coordinates
- model.EnergyFunction
- model.IEnergyFunction
- model.IPositionManager
- model.KnowsConstants
- model.Vector

## Enum Hierarchy

- java.lang.Object
  - java.lang.Enum<E> (implements java.lang.Comparable<T>, java.io.Serializable)
    - model.Pixel.PixelType
    - model.Lattice.Type
    - model.Lattice.State
    - model.IPositionManager.Position
    - model.EnergyFunction.Energy
    - model.Constants.Model
    - model.Cell.CellType
    - model.Cell.Energy
    - model.Cell.State

Skip navigation links


- Overview
- Package
- Class
- Use
- Tree
- Deprecated
- Index
- Help

- Prev
- Next

- Frames
- No Frames

- All Classes
